# Psychological resilience as a bridge between academic work stress and work engagement: evidence from Arab university faculty

**DOI:** 10.3389/fpsyg.2026.1865849

**Published:** 2026-08-05

**Authors:** Benayan Bani Al-Rasheedy, Halima Mohamed Ibrahim Adam, Ahmed Mohammed Ahmed Zayed, Abdalla Sayed Mohamed Gaballa, Mohamed Mohamed Alhadi Hassn Suliman, Hassan Mohamed Riad Mohamed Hassan

**Affiliations:** Psychology Department, Education College, University of Ha’il, Hail, Saudi Arabia

**Keywords:** academic work stress, Arab universities, higher education, PLS-SEM, psychological resilience, work engagement

## Abstract

Academic work in Arab higher education institutions is characterized by a compounding mix of teaching burdens, research expectations, and rigorous quality assurance demands. This study examines how these institutional pressures directly diminish work engagement and evaluate psychological resilience as a protective personal resource. Utilising a sample of 266 faculty members across Jordan, Saudi Arabia, Sudan and Egypt, data were analysed using partial least squares structural equation modelling (PLSA-SEM). The results showed that psychological resilience partially mediated the relationship between academic work stress and work engagement, supporting its role as a core explanatory mechanism. Its moderating effect, however, did not reach statistical significance; this points to resilience functioning more as a stable personal trait that explains the stress-engagement link rather than one that changes depending on how strong or weak resilience is Gender differences in path coefficients were not statistically significant either, although the descriptive pattern showed a stronger negative link between academic work stress and both resilience and engagement among female faculty, and a stronger positive link between resilience and engagement among male faculty. This pattern fits with the added domestic and caregiving expectations placed on women within Arab collectivist family structures. Taken together, the findings broaden the Job Demands-Resources model and Social Cognitive Theory by framing resilience as an explanatory rather than a moderating mechanism in an Arab academic setting, while also supporting practical investment by institutions in resilience-building programs to help sustain faculty engagement under demanding work conditions.

## Introduction

Universities increasingly rely on faculty members to simultaneously fulfil teaching, research, administrative, and community-service responsibilities, creating a work environment characterized by substantial and persistent occupational demands. These demands expose faculty members to significant levels of academic work stress, which may undermine psychological well-being and reduce work engagement (Andijani, 2022). Academic work stress arises from multiple sources, including heavy workloads, time pressure, role ambiguity, and interpersonal demands associated with interactions with colleagues, supervisors, and students (Lee et al., 2022). Unlike many other professions, academic work combines diverse and often competing responsibilities while subjecting faculty members to continuous performance evaluation, thereby generating a uniquely complex and dynamic occupational environment.

Given the pervasive nature of academic work stress, increasing scholarly attention has been directed toward understanding the mechanisms through which faculty members maintain effective functioning and sustained engagement under demanding conditions. Among the personal resources examined in the literature, psychological resilience has emerged as a particularly important protective factor. Psychological resilience refers to the capacity to adapt successfully to adversity, recover from setbacks, and maintain effective functioning despite ongoing stressors (Meng et al., 2023). According to the cognitive model of resilience, resilient individuals employ adaptive cognitive and emotional processes that enable them to respond flexibly to challenges, regulate emotions effectively, and maintain goal-directed behavior under stressful circumstances (Parsons et al., 2016).

The importance of psychological resilience becomes particularly evident in high-demand occupational settings. Research has consistently shown that resilient individuals experience lower levels of stress-related strain and are better able to maintain psychological adjustment when confronted with professional challenges (Parsons et al., 2016; Meng et al., 2023). Furthermore, resilience has been linked to greater persistence, adaptability, and sustained involvement in work-related activities, suggesting that it may play a critical role in preserving work engagement in the face of occupational pressures (Meng et al., 2023).

Work engagement represents a positive, fulfilling work-related state characterized by vigor, dedication, and absorption (Schaufeli et al., 2002). Within academic contexts, engaged faculty members demonstrate higher levels of energy, commitment, and psychological investment in their professional roles. However, sustained exposure to academic work stress may diminish engagement by depleting psychological resources and increasing emotional strain (Bolliger et al., 2020). Consequently, understanding the factors that help faculty members remain engaged despite ongoing occupational pressures has become a significant concern for higher education institutions.

The relationship among academic work stress, psychological resilience, and work engagement can be understood through the Job Demands–Resources (JD-R) framework. According to the JD-R model, excessive job demands contribute to strain and reduced engagement, whereas personal resources help individuals cope with these demands and maintain motivation (Bakker and Demerouti, 2017; Schaufeli and Bakker, 2008). Within this framework, psychological resilience can be conceptualized as a key personal resource that buffers the negative consequences of academic work stress and facilitates sustained engagement. Complementing this perspective, the Conservation of Resources (COR) theory proposes that stress occurs when valued resources are threatened or depleted, whereas personal resources such as resilience help individuals preserve and restore these resources, thereby reducing vulnerability to stress and supporting engagement (Hobfoll et al., 2018; Sonnentag and Meier, 2024).

Empirical evidence supports the role of psychological resilience as a key mechanism linking occupational stress to work engagement. Parsons et al. (2016) and Meng et al. (2023) demonstrated that resilience enables individuals to maintain adaptive functioning and employ effective coping strategies under stressful conditions. Consistent with these findings, evidence from academic and professional settings indicates that resilience contributes to sustained work engagement by helping individuals manage stressors, maintain psychological well-being, and remain invested in their work activities (Bolliger et al., 2020; Meng et al., 2023; Jawhar et al., 2026). Extending this line of research, Zayed et al. (2026) found that psychological resilience contributes to work engagement both directly and indirectly through its relationship with work stress among faculty members. Their findings further suggest that resilience serves as an important explanatory mechanism linking academic work stress to work engagement, highlighting the critical role of personal resources in helping faculty members maintain professional effectiveness and sustained engagement in high-demand academic environments.

Despite growing scholarly interest in academic work stress, psychological resilience, and work engagement, important theoretical and empirical gaps remain. Existing research has largely been conducted in non-academic populations or within Western and non-Arab contexts, limiting understanding of these relationships within Arab higher education institutions. Furthermore, although resilience has been consistently identified as an important personal resource, relatively little attention has been directed toward examining its role as an explanatory mechanism linking academic work stress to work engagement among university faculty members. In addition, the potential influence of gender on these relationships remains insufficiently explored.

### Research gap, research questions, and study objectives

Although a growing body of work has looked at academic work stress, psychological resilience, and work engagement, a few gaps remain open. First, most prior studies have looked at these constructs separately or through fairly simple models, which limits what is known about how academic work stress carries through to work engagement via resilience within one integrated framework. Second, much of the existing evidence comes from students, teachers, healthcare workers, or employees in non-academic and mostly Western settings, so comparatively little is known about how these links play out among faculty in Arab higher education, where teaching, research, administrative, and quality-assurance pressures come together in distinctive ways. Third, while psychological resilience is regularly identified as an important personal resource, few studies have tested its specific function as the mediating bridge between academic work stress and work engagement or examined whether it also moderates the strength of this link. Finally, gender has usually been treated as a control variable rather than a grouping factor capable of revealing meaningful differences in how this stress-resilience-engagement pathway unfolds.

Accordingly, the present study seeks to address these gaps by investigating the role of psychological resilience in the relationship between academic work stress and work engagement among faculty members in Arab universities. Specifically, the study addresses the following research questions:

*RQ1:* Does psychological resilience mediate the relationship between academic work stress and work engagement?

*RQ2:* Does the mediated model differ according to gender (females and males)?

*RQ3:* Does psychological resilience modify the relationship between academic work stress and work engagement?

*RQ4:* Does the moderated model differ according to gender (females and males)?

Guided by these questions, the study aims to:

(1) examine the mediating role of psychological resilience in the relationship between academic work stress and work engagement among faculty members in Arab universities; (2) Determine whether this mediational model differs by gender; (3) explore the Potential moderating role of psychological resilience in the relationship between academic work stress and work engagement; (4) investigate whether any moderating effects vary between male and female participants. These aims are pursued using partial least-squares structural equation modelling (PLS-SEM) to estimate both direct and indirect effects in light of the characteristics of the data and sample.

### Theoretical and conceptual framework

Several theoretical lenses could explain occupational stress and its consequences, among them the Conservation of Resources (COR) theory and the Effort-Reward Imbalance (ERI) model. Yet the present study draws on Social Cognitive Theory (SCT) together with the Job Demands-Resources (JD-R) model, since these two frameworks jointly give the most direct account of how psychological resilience carries the effect of academic work stress through to work engagement. The ERI model centers on the mismatch between employees’ effort and the rewards they receive, making it well suited to stress rooted in perceived inequity, but it says little about the psychological processes that let individuals cope or stay motivated. COR theory, for its part, highlights how resources are gained, protected, or lost, offering a useful account of stress and resilience, yet it does not explain how personal beliefs and self-regulatory processes shape faculty responses to work demands. SCT, by contrast, explains how personal resources such as resilience guide cognitive appraisal, coping, and adaptation, while the JD-R model explains how job demands and personal resources together shape well-being and engagement. Combining SCT and JD-R therefore fits the present study particularly well, since it clarifies both the cognitive mechanisms behind resilience and the organizational pathway through which academic work stress shapes engagement.

Together, Social Cognitive Theory and the JD-R model clarify how psychological resilience acts as the bridge connecting academic work stress to work engagement in university settings. Social Cognitive Theory holds that behavior arises from an ongoing interplay between personal cognition, behavior, and environment; resilience, within this system, shapes how faculty appraise academic demands and choose coping strategies (Bandura, 1997; Schunk and DiBenedetto, 2020). Faculty who see themselves as capable of handling academic pressures tend to read stressors as manageable challenges, keep up their effort, and stay engaged in their professional roles.

The JD-R model adds to this picture by separating job demands, which drain energy, from job resources, which restore and reinvest that energy into work (Bakker and Demerouti, 2017). Here, academic work stress is treated as a core job demand stemming from heavy teaching loads, publication pressure, and administrative duties, while psychological resilience is treated as the personal resource that cushions the negative effects of these demands. When stress runs high and resilience falls short, the model points to a health-impairment process marked by strain, exhaustion, and falling engagement; when resilience is strong, it instead triggers a motivational pathway toward vigor, dedication, and satisfaction (Bakker and Bal, 2010).

Empirical work in educational and occupational contexts supports this dual-process view. Individuals with higher levels of resilience tend to report lower stress and emotional exhaustion, more adaptive emotion regulation, and better psychological adjustment under pressure (Ciarrochi et al., 2015; Dawson and Golijani-Moghaddam, 2020; Hayes et al., 2006). They are also more likely to interpret demanding situations as opportunities for growth, maintain concentration in the presence of competing demands, and persevere when faced with setbacks, all of which are closely aligned with the defining features of work engagement (Gong and Wang, 2023; Schaufeli et al., 2002). Studies have further linked resilience to academic performance, retention, and well-being, suggesting that it plays a role not only in day-to-day coping but also in longer-term educational trajectories (Hsu et al., 2023; Lin et al., 2022; Pepe et al., 2021; Saleem et al., 2022).

Within this framework, psychological resilience sits at the center as the bridge that carries the effect of academic work stress through to work engagement. High stress is expected to erode engagement, but resilience may soften this effect by helping faculty reframe stressors, regulate emotion, and keep investing in academic tasks (Algarni and Alemeri, 2023; Bond et al., 2013; Cabrera-Aguilar et al., 2023). At the same time, sustained engagement may feed back into resilience over time, since experiences of mastery and accomplishment build coping capacity further; this reciprocal possibility fits both Social Cognitive Theory and the JD-R model’s idea of gain spirals, and it also supports treating resilience as a mediator here, while noting that longitudinal designs would be needed to pin down any bidirectional effects. The present study therefore treats psychological resilience mainly as the mediating bridge between academic work stress and work engagement, while also testing whether it exerts a further moderating influence on this relationship.

### Research literature

Recently, the role of work stress, psychological resilience, self-efficacy, and work engagement in educational and professional contexts has been increasingly studied. Yet, results remain fragmented across contexts and populations, revealing the need for a more integrated understanding of the role of personal and organisational resources in adaptation and engagement in demanding work conditions. The present review is structured around three interrelated themes: work stress and psychological resources, self-efficacy and resilience as personal resources, and the role of resilience in fostering work engagement.

#### Theme 1: work stress and psychological resources

The research in educational and occupational settings suggests that the effects of work stress are contingent on the level of stress and on the psychological resources of the individuals. Andijani (2022) found that psychological flexibility reinforced the resilience mechanisms and improved the faculty members’ ability to accept and deal with pressures in the workplace, based on a sample of 93 faculty members from Umm Al-Qura and Al-Baha Universities. Similarly, Marim and Ahmed (2022) stated that resilience defines how people perceive and react to stressful situations and therefore influences their adjustment to psychological demands. Supporting these findings, Jawhar et al. (2026) found that work stress was a significant predictor of emotional exhaustion and psychological distress in a study of 103 nurses and nursing supervisors working in a Lebanese academic medical center. Their findings also point to the idea that ongoing work stress depletes psychological resources and damages well-being. Taken together, these studies indicate that resilience related resources play a protective role against the adverse psychological consequences of occupational stress. However, evidence from Arab higher-education settings remains limited.

#### Theme 2: self-efficacy and resilience as personal resources

Another line of research has emphasized the importance of self-efficacy and resilience as personal resources that facilitate adaptation to demanding work environments. Drawing on data from 455 faculty members in Chinese universities, Liu et al. (2023) found that teaching self-efficacy reduced the negative effects of work pressure on job satisfaction and strengthened professional commitment. Likewise, Heng and Chu (2023), studying English language teachers in China, reported that higher self-efficacy was associated with greater work engagement, persistence, and confidence in managing professional responsibilities. Similar conclusions were reported by Viet et al. (2026), whose study of 1,580 university students demonstrated that self-efficacy enhanced individuals’ capacity for evaluation, analysis, and effective decision-making in high-pressure educational environments. Furthermore, Wang et al. (2024), using a sample of 375 Chinese middle-school English teachers, showed that both resilience and teaching satisfaction mediated the relationship between self-efficacy and well-being. Complementing these findings, Gao et al. (2025) found among 302 Chinese teachers that psychological resilience positively predicted professional identity and sustained commitment to professional activities. Collectively, these findings indicate that self-efficacy and psychological resilience operate as complementary personal resources that facilitate adaptation, promote well-being and enhanced coping under demanding occupational conditions.

#### Theme 3: psychological resilience and work engagement

There is more and more evidence on the direct and indirect role of psychological resilience in work engagement. Cabrera-Aguilar et al. (2023) studied 459 nurses and showed positive relationships between resilience and self-efficacy, and work engagement. Stress, on the other hand, was negatively related to engagement. Additionally, self-efficacy mediated the relationships between resilience-engagement and stress-engagement. Similarly, Wang et al. (2025) found resilience to be an important mediating mechanism between environmental and cognitive factors and enhanced engagement and participation in their study of 606 university EFL students. In a study of 380 teachers, Pepe et al. (2021) found that organisational practices that sought to improve work engagement reduced psychological distress related to reduced job satisfaction. Similarly, Gong and Wang (2023) found that engagement was positively associated with attitudes toward work and career satisfaction among a sample of 221 educators. In line with the above, Algarni and Alemeri (2023) investigated employees’ responses working at a Saudi governmental institution and found that work engagement and job satisfaction significantly improved happiness at work as they were positively associated with organisational commitment. Overall, these findings suggest work engagement as an important outcome of individual and organisational resources and resilience as an important explanatory mechanism linking stressful work environments to positive occupational outcomes. Despite accumulating evidence supporting the positive role of resilience, relatively few studies have examined whether resilience explains the mechanisms through which academic works stress influences work engagement among university faculty, particularly within Arab higher education contexts.

## Methods

### Participants and procedure

Data were drawn from a cross-sectional sample of faculty members recruited from public universities across Jordan, Saudi Arabia, Sudan, and Egypt. The study’s title designates Arab University faculty as the primary population of interest, given that this group constitutes the principal academic workforce in higher education and bears foremost responsibility for teaching, research, and community service-the very domains that generate the occupational demands examined in this study. Faculty assistants were deliberately incorporated into the sampling frame because they occupy institutionally defined academic roles exposing them to structurally comparable demand profiles, encompassing instructional support, research participation, and administrative obligations under analogous workload pressures and performance expectations. Their inclusion therefore extends the ecological validity of the findings without introducing conceptual inconsistency with the study’s title and is consistent with prior organizational research in academic settings wherein teaching and research personnel across rank levels have been treated as a unified population when investigating occupational well-being outcomes (Lee et al., 2022).

The geographic scope of this study was intentionally designed to achieve broad representational coverage of the Arab higher-education landscape rather than to isolate a single national system. The four countries were selected on the basis of a single overarching criterion: the capacity to maximize the number of represented Arab institutions within the available research resources and ethical approval framework, thereby capturing cross-institutional variability in occupational demand structures and enhancing the external validity of the findings beyond any single national context. Within each country, recruitment extended across multiple public universities. In Saudi Arabia, participants were drawn from the universities of Hail, Qassim, Tabuk, Umm Al-Qura, Al-Jouf, and King Faisal. In Egypt, data were collected from the universities of Sohag, Cairo, Ain Shams, Suez Canal, Assiut, Beni Suef, Minia, Helwan, and Fayoum. In Sudan, recruitment included the University of Khartoum and the University of Al-Fasher, among others, and in Jordan, faculty were recruited from public universities accessible through the research team’s institutional networks. The study was intentionally restricted to public universities because they operate under comparable governance structures, unified academic rank systems, and analogous accreditation requirements, ensuring that the occupational demand profiles of participants were structurally equivalent across sites-a prerequisite for valid cross-institutional comparison (Lee et al., 2022). It bears noting that the nationality distribution of the sample reflects participants’ country of origin rather than their employing institution, as a proportion of faculty-particularly in Saudi and Sudanese universities-are nationals of other Arab states holding appointments in those systems, a pattern characteristic of Arab academic labour markets and one that further supports the study’s claim to regional rather than narrowly national representativeness.

Participants were recruited through purposive criterion sampling, whereby eligibility was restricted to individuals holding an active academic appointment-ranging from faculty assistant to full professor-at one of the participating institutions, with a minimum of one completed academic semester in post at the time of data collection. This criterion ensured that all respondents possessed adequate and recent exposure to the occupational demands that constitute the theoretical antecedents of academic work stress as operationalized in this study. Individuals serving in purely administrative capacities without instructional or research duties, as well as those on sabbatical or extended leave during the data collection period, were excluded on the grounds that their occupational circumstances would not reflect the demand structures under investigation. The electronic questionnaire was distributed to 412 eligible faculty members; of these, 266 returned fully completed responses, yielding a response rate of 64.56%, which compares favourably with established benchmarks for online academic surveys (Baruch and Holtom, 2008). To mitigate the risk of common method, bias inherent in single-source self-report data, several procedural safeguards were implemented: full anonymity of responses was assured, item order within each scale was randomized to disrupt halo effects, and participants were explicitly informed that no correct or expected answers existed. A full collinearity assessment conducted within the PLS-SEM framework returned variance inflation factor (VIF) values below 3.3 for all constructs-the threshold recommended by Kock (2015) as indicative of the absence of pathological collinearity attributable to common method variance-providing statistical corroboration that common method bias did not substantively distort the structural estimates reported herein.

All research procedures were conducted in accordance with the ethical principles set forth in the Declaration of Helsinki, and the study received formal approval from the Research Ethics Committee of the authors’ affiliated institution (approval no. H-2024-451). Written informed consent was not required, as data were collected anonymously through a self-administered electronic questionnaire; participants’ consent was considered implicit upon their voluntary completion of the survey. The sample comprised 144 males (54.13%) and 122 females (45.86%). Participants’ ages ranged from under 30 to over 40 years, with the majority falling in the above-40 age group (*n* = 204; 76.69%). Most participants were married (*n* = 203; 76.13%), Egyptian by nationality (*n* = 127; 47.74%), and held doctoral qualifications (*n* = 198; 74.43%). Faculty from education departments accounted for the largest proportion of the sample (*n* = 112; 42.10%). Detailed demographic characteristics are presented in Table 1.

**Table 1:** Participants’ demographic characteristics (*n* = 266—missing zero).

Variable	Frequency	Percent
Sex		
Male	144	54.135
Female	122	45.865
Total	266	100
Age		
<30 years	12	4.511
31–40 years	50	18.797
>40 years	204	76.692
Total	266	100
Nationality		
Jordanian	15	5.639
Saudi	35	13.158
Tunisian	8	3.008
Sudanese	76	28.571
Yemeni	3	1.128
Egyptian	127	47.744
Iraqi	2	0.752
Total	266	100
Academic qualification		
Bachelor’s	11	4.135
Postgraduate diploma	20	7.519
Master’s	37	13.91
Doctorate	198	74.436
Total	266	100
Colleague		
Education	44	16.5
Arts and literature	16	6.0
Sharia and law	10	3.8
Applied	3	1.1
Business administration	12	4.5
Science	4	1.5
Engineering	1	0.4
Computer science and engineering	2	0.8
Nursing	9	3.4
Public health and health informatics	3	1.1
Pharmacy	4	1.5
Medicine	8	3.0
Applied medical sciences	2	0.8
Dental medicine	36	13.5
Other mentions	112	42.1
Total	266	100.0
Marital status		
Single	37	13.91
Married	203	76.316
Divorced	16	6.015
Widowed	10	3.759
Total	266	100

### Measures

In addition to a demographic questionnaire capturing gender, age, educational qualification, nationality, college affiliation, and marital status, three validated instruments were administered.

Psychological resilience was measured using McBride’s (2010) 12-item Psychological Resilience Questionnaire, which assesses an individual’s capacity to manage stress, maintain confidence under adversity, solve problems effectively, and exercise personal agency. Items are rated on a 5-point Likert scale (1 = strongly disagree to 5 = strongly agree). Following PLS-SEM outer loading criteria (Hair et al., 2022), four items were retained (items 6, 9, 11, 12) after items with loadings below the 0.70 threshold were removed. Retained items capture the constructs’ core dimensions: solution-oriented coping, internal locus of control, stress regulation, and confidence in one’s role.

Academic work stress was assessed using a researcher-developed scale constructed to address the unique demand profile of university faculty members, covering five subdimensions: general psychological stress, research and publication pressures, teaching burden, administrative demands, and quality-assurance expectations. The 18-item scale uses a 5-point Likert format (1 = strongly disagree to 5 = strongly agree). Four items demonstrated acceptable psychometric properties in the PLS-SEM analysis (items 1, 2, 16, 17) and were retained for structural estimation.

Work engagement was measured using Schaufeli et al.'s (2002) Utrecht Work Engagement Scale (UWES-17), which operationalizes engagement through three dimensions: vigour, dedication, and absorption. The response scale ranges from 1 (never) to 5 (always). Eight items achieved outer loadings ≥ 0.70 (items 1, 2, 3, 4, 5, 7, 9, 11) and were retained. Scales were translated into Arabic using a forward-backward translation procedure. An initial translation was produced independently by two bilingual psychologists, reconciled through discussion, and then back-translated into English by a third bilingual expert unacquainted with the original instruments. Discrepancies between the original and back-translated versions were resolved through iterative revision until semantic equivalence was confirmed. This procedure is consistent with established cross-cultural adaptation guidelines (Beaton et al., 2000).

The two standardized instruments-McBride’s (2010) psychological resilience scale and Schaufeli et al.'s (2002) work engagement scale-were originally developed in English and adapted for Arabic-speaking academic populations through a systematic forward-backward translation procedure (Beaton et al., 2000). Two bilingual psychologists independently produced Arabic translations of each scale, prioritizing conceptual fidelity over literal correspondence, before reconciling their versions through structured discussion. A third bilingual expert, unacquainted with the original instruments, then back-translated the reconciled Arabic text into English, and any semantic discrepancies identified through comparison with the originals were resolved iteratively until equivalence was confirmed. Certain items required contextual rewording to align with the structural realities of Arab academic environments; for instance, engagement items were rendered using Arabic lexical choices that more precisely convey sustained professional investment rather than transient affective states. The Academic Work Stress Scale, by contrast, was developed directly in Arabic by the research team to capture the specific demand configurations characteristic of Arab University contexts-encompassing teaching load, research and publication pressures, administrative obligations, and quality-assurance expectations-thereby circumventing the need for cross-language adaptation while ensuring face and content validity for the target population from the outset. All three instruments were subsequently piloted with ten faculty members external to the main study, whose feedback informed minor wording refinements prior to final deployment.

Although, the removal of indicators with low outer loadings improved measurement reliability and construct validity in accordance with established PLS-SEM guidelines. Hair et al. (2022), it may have reduced the breadth of construct representation. This issue is revisited in the limitations section.

### Statistical analysis

The researchers employed Partial Least Squares Structural Equation Modelling (PLS-SEM) in preference to Covariance-Based Structural Equation Modelling (CB-SEM), a choice grounded in methodological and theoretical considerations specific to the present study. Unlike CB-SEM, which is optimally suited to confirmatory theory testing within fully specified, theoretically mature models requiring large samples and multivariate normality, PLS-SEM is a distribution-free composite-based estimator particularly well-suited to prediction-oriented research objectives and to models incorporating interaction terms-such as the moderated mediation pathway tested here-whose non-normal distributional properties are well-documented (Hair et al., 2022; Henseler et al., 2016). A central aim of this study is to evaluate the explanatory capacity of psychological resilience as a mechanism linking occupational stress to work engagement, as indexed by R2 and f^2^ metrics; this predictive orientation aligns more naturally with PLS-SEM’s analytical architecture than with CB-SEM’s emphasis on reproducing observed covariance structures. Moreover, given that the structural relationships examined here-particularly the simultaneous mediation and moderation by psychological resilience within an Arab academic context-have not been comprehensively investigated in prior literature, the model occupies an exploratory-confirmatory rather than purely replicative theoretical position, a level of theoretical maturity for which PLS-SEM is methodologically preferred (Hair et al., 2019). Collectively, these considerations justify the selection of PLS-SEM as the analytical method of choice for the present study.

Data analysis was conducted using SmartPLS 4, following a two-stage PLS-SEM procedure (Hair et al., 2022). In the first stage, the measurement model was evaluated through assessment of outer loadings, internal consistency reliability (Cronbach’s *α* and composite reliability), convergent validity (average variance extracted, AVE), and discriminant validity (HTMT ratios and Fornell-Larcker criterion). Indicators with outer loadings below 0.70 were removed to optimize construct validity (Sarstedt et al., 2022). In the second stage, the structural model was estimated using a bootstrapping procedure with 5,000 resamples to derive path coefficients and their 95% confidence intervals. Effect sizes (f^2^) were computed to assess the practical significance of structural paths, and the moderated mediation hypothesis was tested via the product-indicator approach. Multigroup analysis by gender was conducted to examine whether structural paths differed between male and female subsamples. Model fit was evaluated using SRMR and NFI indices.

### Reliability and validity

Reliability was supported by Cronbach’s α values ranging from 0.746 to 0.921 and composite reliability (ρ_c) values between 0.839 and 0.936, all exceeding the 0.70 criterion (Hair et al., 2019). Convergent validity was confirmed by AVE values between 0.567 and 0.645, surpassing the 0.50 threshold (Fornell and Larcker, 1981), and by statistically significant outer loadings ranging from 0.710 to 0.858. Discriminant validity was established via HTMT ratios (0.436–0.531, all < 0.85) and the Fornell-Larcker criterion, wherein each construct’s AVE square root (0.753–0.803) exceeded its inter-construct correlations. Cross-loadings further confirmed that every item loaded more strongly on its assigned construct than on any other. Full model statistics are presented in Tables 2, 3.

**Table 2:** Model and item paths, loadings, t-values, statistical significance, variance inflation (VIF), and reliability coefficients.

Paths	Items	Outer loadings	T-statistics (|O/STDEV|)	*p*-values	VIF	Cronbach’s alpha	Composite reliability (rho_a)	Composite reliability (rho_c)	Average variance extracted (AVE)
Occup_str1 ← occupational stress	I feel very burdened with teaching academic courses to students.	0.811	21.84	0.000	1.721	0.800	0.815	0.867	0.620
Occup_str16 ← occupational stress	Sometimes when I think about my work, I feel a tightness in my chest.	0.749	13.885	0.000	2.485				
Occup_str17 ← occupational stress	Often, my work becomes a great burden.	0.778	16.21	0.000	2.583				
Occup_str2 ← occupational stress	I have a lot of quality work, and I am afraid I have little time to do it.	0.809	24.434	0.000	1.762				
Resilience11 ← psychological resilience	I manage my stress levels well.	0.789	22.06	0.000	1.655	0.746	0.751	0.839	0.567
Resilience12 ← psychological resilience	I feel confident and secure in my position.	0.789	21.933	0.000	1.575				
Resilience6 ← psychological resilience	I am good at finding solutions to problems.	0.710	10.703	0.000	1.397				
Resilience9 ← psychological resilience	I try to control events rather than being a victim of circumstances.	0.722	17.295	0.000	1.287				
work_eng1 ← work engagement	At work, I feel full of energy.	0.813	34.094	0.000	2.377	0.921	0.926	0.936	0.645
work_eng11 ← work engagement	I love my work, and I am immersed in it.	0.858	38.379	0.000	2.866				
work_eng2 ← work engagement	My work is useful and purposeful.	0.756	17.373	0.000	2.070				
work_eng3 ← work engagement	Time flies when I work.	0.762	17.317	0.000	2.121				
work_eng4 ← work engagement	I feel strong and energetic in my work.	0.837	26.141	0.000	2.861				
work_eng5 ← work engagement	Enthusiastic about my work.	0.841	21.224	0.000	2.928				
work_eng7 ← work engagement	My work inspires me.	0.790	23.083	0.000	2.336				
work_eng9 ← work engagement	I feel happy when I work hard.	0.759	18.393	0.000	2.079				

**Table 3:** Cross-loading of items with their constructs.

Items	Occupational stress	Psychological resilience	Work engagement
Occup_str1	0.811	−0.355	−0.363
Occup_str16	0.749	−0.196	−0.28
Occup_str17	0.778	−0.198	−0.293
Occup_str2	0.809	−0.327	−0.306
Resilience11	−0.288	0.789	0.311
Resilience12	−0.262	0.789	0.386
Resilience6	−0.208	0.710	0.284
Resilience9	−0.302	0.722	0.354
work_eng1	−0.461	0.396	0.813
work_eng11	−0.373	0.413	0.858
work_eng2	−0.26	0.34	0.756
work_eng3	−0.217	0.337	0.762
work_eng4	−0.368	0.343	0.837
work_eng5	−0.303	0.341	0.841
work_eng7	−0.283	0.361	0.790
work_eng9	−0.225	0.327	0.759

## Results

### Model fit

Model fit was verified by the standardized root-mean-squared-residual (SRMR) and the normed fit index (NFI). SRMR was 0.075. According to Hu and Bentler (1999), a value of <0.08 is generally considered a good fit. The NFI value reached 0.796, which is close to the recommended cutoff of 0.80 and suggests a moderate level of fit. Furthermore, the additional indices (d_G = 0.291 and d_ULS = 0.764) provide further evidence supporting the stability of the model.

### Mediated moderated model

**Figure 1:**
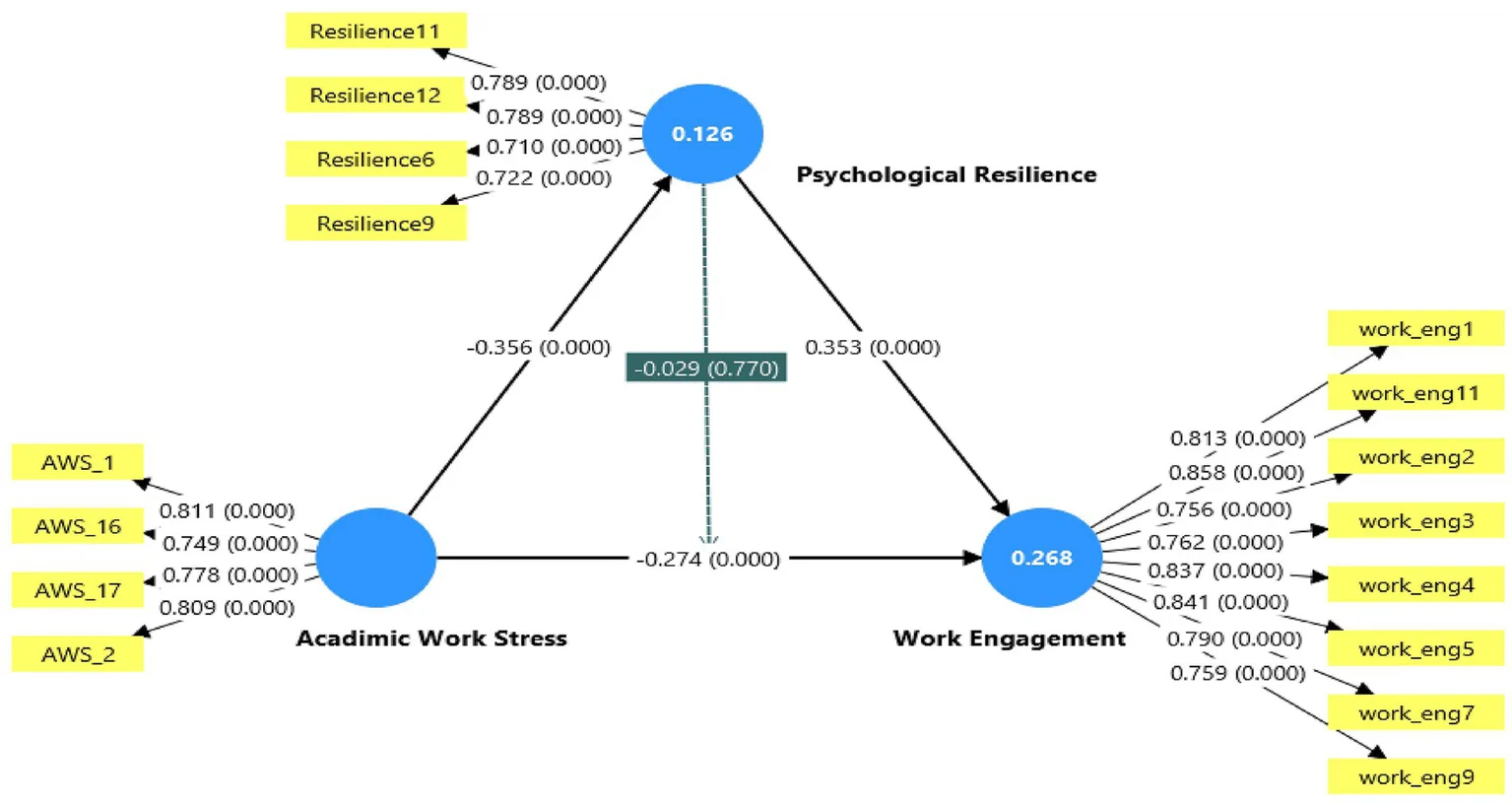
PLS-SEM complete mediated moderated model.

To detect the mediating and moderating effects of the hypothesized model, a multiple PLS-SEM algorithm analysis was conducted between two groups for the gender variable (males and females), in addition to verifying its measurement stability. Figure 1 depicts the overall model, and Table 4 presents the direct and indirect effects of the overall model and gender models.

**Table 4:** Direct and indirect effects, t-value and its significance for the total, female, and male models.

Path coefficients	Original sample (O)	T-statistics (|O/STDEV|)	*p*-values	0.025	0.975	Decision
Total model						
Direct effects						
Occupational stress → Psychological resilience	−0.356	5.767	0.000	−0.462	−0.215	Accepted
Occupational stress → work engagement	−0.274	3.642	0.000	−0.404	−0.111	Accepted
Psychological resilience → work engagement	0.353	4.159	0.000	0.187	0.513	Accepted
Psychological resilience x occupational stress → work engagement	−0.029	0.289	0.773	−0.204	0.179	
Indirect effects						
Occupational stress → work engagement	−0.125	4.201	0.000	−0.187	−0.07	Accepted
Female model						
Direct effects						
Occupational stress → Psychological resilience	−0.464	5.829	0.000	−0.596	−0.278	Accepted
Occupational stress → work engagement	−0.358	4.248	0.000	−0.501	−0.167	Accepted
Psychological resilience → Work engagement	0.247	2.554	0.011	0.021	0.41	Accepted
Psychological resilience x Occupational stress → work engagement	−0.045	0.337	0.736	−0.261	0.254	
Indirect effects						
Occupational stress → work engagement	−0.114	2.48	0.013	−0.199	−0.014	Accepted
Male model						
Direct effects						
Occupational stress → Psychological resilience	−0.278	3.491	0.000	−0.393	−0.06	Accepted
Occupational stress → work engagement	−0.244	2.554	0.011	−0.429	−0.074	Accepted
Psychological resilience → Work engagement	0.478	4.325	0.000	0.254	0.659	Accepted
Psychological resilience x Occupational stress → work engagement	0.072	0.802	0.423	−0.12	0.224	
Indirect effects						
Occupational stress → work engagement	−0.133	2.974	0.003	−0.217	−0.052	Accepted

### Coefficients of determination (R^2^)

The coefficient of determination pertaining to occupational stress was found to be R^2^ = 0.126 (adjusted = 0.123), which signifies a limited capacity for explanation.

Conversely, work engagement attained a coefficient of R^2^ = 0.267 (adjusted = 0.261), indicating a more robust explanatory power and demonstrating the model’s efficacy in elucidating a significant fraction of variance associated with this construct.

### Effect sizes (f^2^) and their substantive interpretation

Effect size values (f^2^) were computed to assess the practical significance of each structural path, complementing the statistical significance of the path coefficients. For the path from occupational stress to work engagement, f^2^ = 0.090 indicates a small-to-moderate effect, suggesting that while occupational stress independently reduces engagement, its direct influence-once psychological resilience is accounted for-does not dominate the structural model. This pattern implies that occupational stress alone is an insufficient predictor of disengagement and that its impact is channelled substantially through psychological resources rather than operating in isolation. From a practical standpoint, interventions targeting stress reduction may yield only modest direct gains in engagement unless they simultaneously strengthen resilience. The effect of psychological resilience on occupational stress yielded f^2^ = 0.145, a moderate effect that indicates resilience exerts a meaningful suppressive influence on how faculty experience and appraise work-related demands. This finding is consistent with Conservation of Resources theory (Hobfoll et al., 2018), which holds that personal resources-of which resilience is a primary exemplar-function as protective buffers that reduce the subjective weight of environmental demands. Faculty members with greater resilience appear to perceive the same objective workload and performance pressures as less taxing, which in turn preserves their cognitive and affective capacity for engagement. Similarly, the effect of psychological resilience on work engagement produced f^2^ = 0.146, also a moderate effect, underscoring that resilience is not merely a stress buffer but an active driver of motivational investment in academic work. This aligns with the JD-R model’s motivational pathway (Bakker and Demerouti, 2017), wherein personal resources energize engagement by enabling individuals to extract meaning and sustain effort even under demanding conditions. Collectively, the two moderate f^2^ values associated with psychological resilience-one on the antecedent (stress) and one on the outcome (engagement)-empirically substantiate its pivotal mediating position in the structural model and indicate that resilience contributes meaningful explanatory variance to both sides of the mediation pathway, not merely serving as a statistical intermediary.

### Path coefficients

Occupational stress → Psychological resilience: −0.356, exhibiting statistical significance (*p* < 0.001).

Occupational stress → Work engagement: −0.274, demonstrating statistical significance (*p* < 0.001).

Psychological resilience → Work engagement: 0.353, showing statistical significance (*p* < 0.001).

The indirect effect of occupational stress on work engagement through psychological resilience was calculated at −0.125, showing statistical significance (*p* < 0.001).

### Gender-based differences

#### Female model

Occupational stress exerted a more pronounced negative influence on psychological resilience at −0.464 and on work engagement at −0.358. Psychological resilience exerted a positive effect on work engagement at 0.247; however, this effect was comparatively weaker than that observed in males. The indirect pathway linking occupational stress to work engagement through resilience was determined to be −0.114, which is statistically significant.

#### Male model

Occupational stress exerted a negative influence on psychological resilience at −0.278 and on work engagement at −0.244, albeit with a less pronounced effect than that observed in females. Psychological resilience demonstrated a substantial positive effect on work engagement at 0.478, exceeding the effect noted in females. The indirect effect of occupational stress on work engagement via resilience was −0.133, which is statistically significant.

The paths of the overall model revealed that the direct effects were significant for all paths, the female model, and the male model, as shown in Table 4. Indirect effects were also significant; however, all interaction paths in the three models were insignificant: psychological resilience x occupational stress à work engagement, indicating the presence of the partial mediation of psychological resilience. No moderating effect was observed in the relationship between academic work stress and work engagement. The analyses revealed gender-related variations in the strength of associations but these did not reach statistical significance, which calls for caution when interpreting gender differences. This outcome indicates that resilience operates as a partial mediator rather than a moderator of the relationship. Therefore, psychological resilience partially mediated the relationship between academic work stress and work engagement in the overall, female, and male models. Neither psychological resilience nor gender moderated this relationship. This indicates that resilience played a mediating role in the relationship between academic work stress and work engagement. Furthermore, no differences were observed between males and females, as both used psychological resilience to overcome academic work stress.

## Discussion

The findings of the present study underscore the pivotal role of psychological resilience in explaining the relationship between academic work stress and work engagement. In the following sections, these findings are interpreted considering relevant theoretical perspectives and empirical evidence, and their implications for academic settings are discussed.

### Psychological resilience as a partial mediator

The results provide robust evidence that psychological resilience partially mediates the relationship between academic work stress and work engagement, a pattern that remained consistent across all tested models. This finding is consistent with previous research identifying psychological resilience as a critical personal resource that mitigates the adverse effects of stress while promoting adaptive functioning and sustained work engagement in demanding professional environments (Cabrera-Aguilar et al., 2023; Meng et al., 2023; Jawhar et al., 2026; Zayed et al., 2026). The stability of the indirect effect across models, despite the absence of significant moderation by either resilience or gender, suggests that resilience functions as a fundamental explanatory mechanism through which academic work stress influences work engagement rather than as a context-dependent or conditional factor. These findings reinforce the view that resilience not only protects individuals from the detrimental consequences of occupational stress but also supports the maintenance of engagement in the face of persistent professional demands.

### Gender differences in structural paths

The multigroup analysis pointed to differences in path magnitude between male and female faculty, though these differences fell short of statistical significance and should be read with caution. Descriptively, academic work stress showed a stronger negative effect on resilience among female faculty (*β* = −0.464) compared with male faculty (β = −0.278), while resilience showed a stronger positive link with work engagement among male faculty. This mirrors what Zayed et al. (2026) reported in a comparable Arab faculty sample and may reflect a broader regional pattern in which women carry a heavier cumulative load of institutional demands, leaving less room for resilience to translate into engagement. Still, because the indirect path from stress to engagement through resilience held up as significant in both groups, resilience appears to work as a shared explanatory mechanism for faculty regardless of gender, even as its relative strength varies somewhat between the two.

### Theoretical integration

The current results are in line with the Job Demands–Resources (JD-R) model that considers academic work stress as a job demand that depletes employees’ psychological energy and might diminish work engagement when sufficient resources are not available (Bakker and Demerouti, 2017). Psychological resilience as an important personal resource in the present study alleviated the negative impact of academic work stress on engagement. This finding supports the JD-R proposition that personal resources help individuals to cope with demanding work conditions and maintain motivation even in the face of adverse circumstances (Bakker and Demerouti, 2017; Bakker and Bal, 2010). In addition, the gender differences found suggest that the balance of demands and resources may differ across groups. Although the observed gender related differences should be interpreted cautiously, the descriptive pattern of results suggests that female faculty members may experience greater exposure to competing professional and social role demands, whereas resilience appeared to demonstrate a relatively stronger association with work engagement among male participants.

The findings also contribute to Social Cognitive Theory (Bandura, 1997) by emphasising the role of psychological resilience as an adaptive self-regulatory process that enables engagement in stressful conditions. SCT proposes that people’s beliefs about their abilities influence their cognitive evaluations, behavioral reactions and perseverance when faced with difficulties (Bandura, 1997; Schunk and DiBenedetto, 2020). Given the close conceptual relationship between resilience and self-efficacy, resilient faculty members may be more likely to reframe academic stressors as manageable challenges rather than overwhelming hurdles. This interpretation is consistent with evidence indicating that self-efficacy and resilience jointly enhance adaptation, persistence, and professional functioning in demanding educational environments (Jeffords et al., 2020; Viet et al., 2026).

The current findings are also consistent with Conservation of Resources (COR) theory, which posits that individuals strive to acquire, protect, and maintain valued personal resources, and that stress arises when these resources are threatened or lost (Hobfoll et al., 2018). In addition to supporting JD-R and SCT. The negative association between academic work stress and engagement observed in the current study may be indicative of a resource-loss process, whereas the positive role of resilience lends support to the idea that personal resources can break spirals of loss and promote resource-gain cycles that maintain engagement (Hobfoll et al., 2018; Sonnentag and Meier, 2024). Similarly, although the Effort–Reward Imbalance (ERI) model emphasises the detrimental effects of a mismatch between efforts made at work and rewards received, the present results suggest that resilience could help to explain the individual differences in engagement and adaptation to similar academic demands.

The explanatory role of psychological resilience observed in the present study partly reflect characteristics of the Arab higher education context. In this region, Individual resilience does not function in isolation; deeply reinforced by collectivistic social values, close-knit family support networks, and cultural and religious practices (Alamri and Al-Abyadh, 2024). Specifically, Islamic spiritual coping mechanisms such as patience, perseverance, and meaning-making in the face of adversity are widely recognized as sources of psychological strength. Faculty members have a cognitive reframing framework. This localized wisdom allows them to view intense institutional demands not merely as arbitrary stressors, but as meaningful professional duties, thereby preserving their work engagement. Regarding generalizability, these findings are highly applicable to other academic systems in the Middle East and North Africa that share similar collectivistic structures and quality assurance pressures. However, caution should be exercised when applying these results to highly individualistic Western academic environments. Resilience pathways are often tied to individual autonomy and distinct institutional reward systems rather than collective or spiritual coping mechanisms. Because cultural values were not directly measured in the present study, these interpretations should be regarded as theoretically informed explanations rather than empirically verified conclusions and should be examined in future cross-cultural research.

Together, the study contributes to the extant literature by integrating the organisational mechanisms emphasised in the JD-R model with the self-regulatory processes emphasised by Social Cognitive Theory. In doing so, the study provides empirical support for psychological resilience not only as a buffer against occupational stress, but also as an important explanatory mechanism linking academic work stress to work engagement, particularly within the underexplored context of Arab higher education.

### Practical implications

The present findings have important implications for university administrators, faculty development centres, human resource units and higher education policy makers. Because psychological resilience demonstrated a significant mediating effect between academic work stress and work engagement, interventions that strengthen resilience may represent an effective strategy for maintaining faculty engagement under demanding academic conditions. Psychological resilience emerged as a key factor in sustaining work engagement under demanding academic conditions, so interventions should target both individual and organisational resources. At the individual level, resilience-enhancing initiatives could be particularly useful for faculty experiencing high role strain, especially early-career academics and female faculty. Examples include mindfulness-based programmes, mentoring schemes, adaptive coping workshops and interventions to reduce work–family conflict. These approaches are consistent with evidence that resilience-related resources enhance coping capacity and sustain engagement in challenging circumstances (Parsons et al., 2016; Freire et al., 2020; Fletcher and Sarkar, 2013).

At the institutional level, workload-management policies aimed at reducing excessive teaching and administrative responsibilities while increasing access to supportive resources should be considered. Previous evidence shows that autonomy and supervisory support enhance an employee’s well-being and reduce stress, but too much workload hampers adaptation and work-life balance (Zaki et al., 2025; Bakker and Demerouti, 2017). Also, recent findings in academic and healthcare settings suggest that preventive interventions addressing stress reduction and burnout prevention may enhance psychological well-being and reduce the adverse effects of occupational stress (Jawhar et al., 2026).

Universities might also benefit from resource-oriented systems that provide timely feedback, peer-support opportunities and structured professional development programmes. Consistent with JD-R and COR perspectives, such resources can support faculty members in transforming demanding work conditions into opportunities for growth and sustained engagement, rather than sources of chronic strain (Bakker and Demerouti, 2017; Hobfoll et al., 2018). Given prior research suggesting that early career faculty and those with limited institutional support are particularly vulnerable to decreases in self-efficacy, resilience, and engagement (Jeffords et al., 2020), special attention should be paid to these groups.

### Recommendations

Based on the findings of the present study, some recommendations can be presented. First, resilience-development programmes need to be embedded in faculty training and professional development efforts at universities. Second, institutional policies should aim at reducing excessive administrative burden and improving workload distribution. Thirdly, mentoring programs and peer-support systems should be strengthened to improve personal and organisational resources. Fourth, universities should support programs for faculty members that promote work-life balance, psychological well-being, and effective coping strategies. Finally, future studies are encouraged to examine longitudinal relationships among academic work stress, resilience and work engagement, and explore other personal and organisational resources, such as self-efficacy, psychological flexibility, leadership support and organisational climate.

## Conclusion

The findings of this study demonstrate that psychological resilience plays a significant role in explaining the relationship between academic work stress and work engagement among faculty members in Arab universities. Psychological resilience partially mediated the negative association between academic work stress and work engagement, highlighting its function as a key explanatory mechanism through which faculty members maintain engagement under demanding academic conditions. While descriptive patterns indicated slightly higher vulnerability to stress among female faculty, multi-group analysis confirmed that structural pathways did not differ significantly by gender, demonstrating that both male and female academics rely fundamentally on resilience to navigate institutional demands. By focusing on the unique context of Arab higher education, this study advances our understanding of personal resilience and its contribution to occupational well-being, underscoring that institutional policies must actively protect faculty resources to sustain long-term engagement.

### Limitations

Although our research yielded important results, a few limitations should be noted. First, this study used self-reported measures, which are prone to social desirability or response style biases. Future studies should use multimethod approaches, such as observational data or physiological measures, to improve the generalizability of the findings. Second, this study examined psychological resilience as the primary mediator; however, the role of additional mediating variables in the association between academic work stress and work engagement requires further investigation. Additional variables, such as social support, coping strategies, and individual differences in personality traits, might also substantially influence these dynamics. Exploring these and other contexts will yield a richer picture of the complications of academic work. Third, the elimination of items with loadings below 0.70, although a methodological practice that strengthens construct validity and reliability, resulted in a reduction of certain explanatory indicators.

Moreover, it is important to recognize that cross-sectional data, as used in this study, can limit causal interpretations, and longitudinal or experimental designs may provide deeper insights into the temporal relationships among variables. The sample was also drawn from a specific academic environment, which may constrain the applicability of these findings to broader or more diverse educational contexts. Future research should consider larger and more heterogeneous samples, incorporating advanced psychometric analyses such as measurement invariance testing across groups to establish the robustness of the instruments used. Finally, while the current study focused on quantitative measures, integrating qualitative approaches—such as in-depth interviews or focus groups—could capture nuanced perspectives and contextual factors that quantitative data alone may overlook.

## Data Availability

The original contributions presented in the study are included in the article/supplementary material, further inquiries can be directed to the corresponding author.
